# *Spatholobus suberectus* Ameliorates Diabetes-Induced Renal Damage by Suppressing Advanced Glycation End Products in db/db Mice

**DOI:** 10.3390/ijms19092774

**Published:** 2018-09-14

**Authors:** Moon Ho Do, Jinyoung Hur, Jiwon Choi, Yoonsook Kim, Ho-Young Park, Sang Keun Ha

**Affiliations:** 1 Korea Food Research Institute, 245, Nongsaengmyeong-ro, Iseo-myeon, Wanju_Gun, Jeollabuk-do 55365, Korea; Do.Moon-ho@kfri.re.kr (M.H.D.); jyhur@kfri.re.kr (J.H.); Choi.Ji-won@kfri.re.kr (J.C.); kimyus@kfri.re.kr (Y.K.), hypark@kfri.re.kr (H.-Y.P.); 2 Divison of Food Biotechnology, University of Science and Technology, Daejeon 305350, Korea

**Keywords:** advanced glycation end products (AGEs), diabetic nephropathy, nuclear factor erythroid 2-related factor 2 (Nrf2), glyoxalase 1 (Glo1), *Spatholobus suberectus*

## Abstract

*Spatholobus suberectus* (SS) is a medicinal herb commonly used in Asia to treat anemia, menoxenia and rheumatism. However, its effect of diabetes-induced renal damage and mechanisms of action against advanced glycation end-products (AGEs) are unclear. In this study, we evaluated the effects of SS on diabetes-induced renal damage and explored the possible underlying mechanisms using db/db type 2 diabetes mice. db/db mice were administered SS extract (50 mg/kg) orally for 6 weeks. SS-treated group did not change body weight, blood glucose and glycated hemoglobin (HbA1c) levels. However, SS treatment reversed diabetes-induced dyslipidemia and urinary albumin/creatinine ratio in db/db mice. Moreover, SS administration showed significantly increased protein expression of nuclear factor erythroid 2-related factor 2 (Nrf2), which is a transcription factor for antioxidant enzyme. SS significantly upregulated glyoxalase 1 (Glo1) and NADPH quinine oxidoreductase 1 (NQO1) expression but reduced CML accumulation and downregulated receptor for AGEs (RAGE). Furthermore, SS showed significant decrease of periodic acid–Schiff (PAS)-positive staining and AGEs accumulation in histological and immunohistochemical analyses of kidney tissues. Taken together, we concluded that SS ameliorated the renal damage by inhibiting diabetes-induced glucotoxicity, dyslipidemia and oxidative stress, through the Nrf2/antioxidant responsive element (ARE) stress-response system.

## 1. Introduction

Diabetes mellitus is one of the most complicated diseases; however, current knowledge about its cause remains limited. Long-term hyperglycemia in diabetes mellitus leads to secondary complications such as heart disease, stroke, retinopathy, nephropathy, and neuropathy [1]. Among them, diabetic nephropathy is one of the most common complications of diabetes and the leading cause of end-stage renal disease through several pathologic pathways [2]. It is well-known that renal cell loss in early diabetic nephropathy can be caused by hyperglycemia, oxidative stress, and formation of advanced glycation end products (AGEs) [3].

AGEs can be formed by non-enzymatic reactions between reducing sugars and amino groups of proteins. AGEs-induced cell hypertrophy, a condition caused by long-term hyperglycemia, was observed in the kidney or renal cells such as renal tubular cells, podocytes and glomerular mesangial cells, and then it led to diabetic nephropathy [4]. Suzuki et al. reported that strongly stained AGEs and mesangial expansion are observed in patients with diabetic nephropathy [5]. AGEs-induced chronic inflammation and the consequent cellular and tissue impairment are triggered by increasing oxidative stress and various inflammatory or pro-apoptotic cytokines via an interaction between AGEs and the receptor for AGEs (RAGE) [6]. Recently, some of ACCORD studies found glycemic control did not effective to prevent diabetic complications [7,8]. For these reason, Moraru et al. suggested that insulin resistance and hyperglycemia are result from the disease, but are not part of the causal cascade leading to type 2 diabetes [9]. Studies reported that inhibition of AGEs formation or accumulation in diverse tissues is effective strategy to protect against diabetes and diabetic complications [10]. Moreover, many AGEs-targeted drug candidates ameliorated diabetic complications without changes of blood glucose [11,12]. Therefore, ameliorating of AGEs-induced glucotoxicity might be an important to treatment of diabetic complications. 

The glyoxalase system comprises glyoxalase 1 (Glo1) and glyoxalase 2 and is known to play an important role in detoxification of AGEs. Glo1 overexpression causes detoxification of hyperglycemia-induced carbonyl and oxidative stress and inhibits AGEs formation [13]. Nuclear factor erythroid 2-related factor 2 (Nrf2) is well-known as a regulator of Glo1 and antioxidant enzymes such as NADPH quinine oxidoreductase 1 (NQO1), heme oxygenase 1, and superoxide dismutase [14]. Thus, activation of Nrf2 and Glo1 could be an effective strategy for treating diabetic complications.

*Spatholobus suberectus* (SS), belonging to the Leguminosae family (Fabaceae), is a widely used traditional medicine for the treatment of anemia, menoxenia, and rheumatism [15]. Component analysis of *S*. *suberectus* showed that the herb contains various types of phenolic compounds, including flavones, isoflavonoids, flavanones, flavanonols, and chalcones [16]. The herb has been shown to have diverse benefits including anti-inflammatory, antioxidant, and antirheumatic effects [17]. Moreover, Zhao et al. demonstrated that *S*. *suberectus* had a protective effect against streptozotocin-induced diabetes in a mouse model [18]. Nonetheless, no study has investigated the anti-AGEs activity and protective effect of *S*. *suberectus* against diabetic nephropathy and the underlying mechanisms. 

We performed an in vitro screening to determine the inhibitory effects or breaking ability of several natural products against high glucose and fructose conditions. Among them, *S*. *suberectus* showed effective anti-AGEs activity. Moreover, db/db mouse is a well-known type 2 diabetes mouse model and shows diverse nephropathy symptoms such as mesangial expansion, thickening of the glomerular basement membrane and albuminuria [19]. Therefore, we investigated its effects in a diabetes mouse model and evaluated its protective effects against diabetes indices and renal disorders associated with AGEs.

## 2. Results

### 2.1. Formononetin Concentration in S. suberectus 

**Figure 1:**
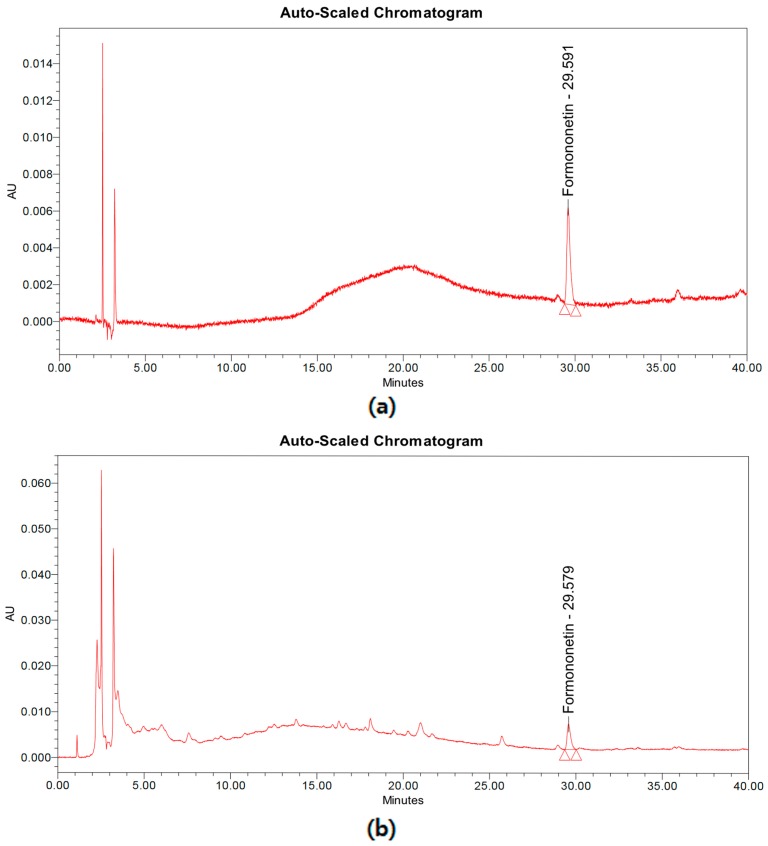
Chromatogram of formononetin and *Spatholobus suberectus* extract. (**a**) Chromatogram of formononetin standard compounds. (**b**) Chromatogram of *S. suberectus* extract.

SUN et al. reported that sixteen components have been detected from *Spatholobus suberectus* [20]. Among these compounds, formononetin is highly contained in *S*. *suberectus* and it is well-known to possess various pharmacological effects. The high-performance liquid chromatography (HPLC) analysis revealed the concentration of formononetin in *S*. *suberectus* to be 1.106 ± 0.021 mg/g (Figure 1), and the linearity of formononetin standard compound was found to be *r*^2^ = 0.999. 

### 2.2. Effect of S. suberectus Extract on AGEs Formation and Breaking 

**Figure 2:**
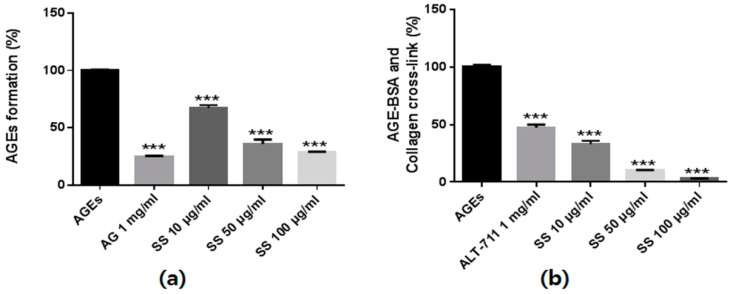
The anti-glucotoxicity effect of *Spatholobus suberectus*. (**a**) The inhibitory effects of *S. suberectus* extract on in vitro AGEs formation. Bovine serum albumin (5 mg/mL) was incubated with 25 mM glucose and fructose in the presence or absence of *S*. *suberectus* in phosphate-buffered saline for 14 days. (**b**) The ability of *S*. *suberectus* to break AGEs cross-linking. The optical density of AGEs-collagen cross-linking was determined using tetramethylbenzidine as the substrate. Aminoguanidine (AG) and alagebrium (ALT-711) were used for positive control of AGEs formation and AGEs cross-linking assay. Each experiment is performed three independent experiments for three repeat experiment, and data are presented as the mean ± standard deviation (SD, %) values (* *p* < 0.05 and *** *p* < 0.001 vs. AGEs).

We investigated the inhibitory effects of the *S*. *suberectus* extract on AGEs formation based on fluorescence excitement and emission spectra at 350 nm and 450 nm, respectively. As shown in Figure 2, bovine serum albumin (BSA) incubation with glucose and fructose significantly induced AGEs formation. However, *S*. *suberectus* treatment inhibited AGEs formation in a dose-dependent manner. 

To investigate the AGEs-collagen cross-link–breaking ability of *S*. *suberectus*, we determined the strength of cross-link breaking using an enzyme-linked immunosorbent assay (ELISA). The AGEs-BSA treatment markedly increased the AGEs-BSA–induced cross-links to collagen. However, treatment with the *S*. *suberectus* extract markedly broke AGEs-collagen cross-linking in a dose-dependent manner (Figure 2b). Taken together, these results indicate that *S*. *suberectus* has not only an inhibitory effect on AGEs formation but also has the ability to break AGEs-collagen cross-linking.

### 2.3. Effects of the S. suberectus Extract on the Diabetes Parameters and Lipid Profiles 

**Figure 3:**
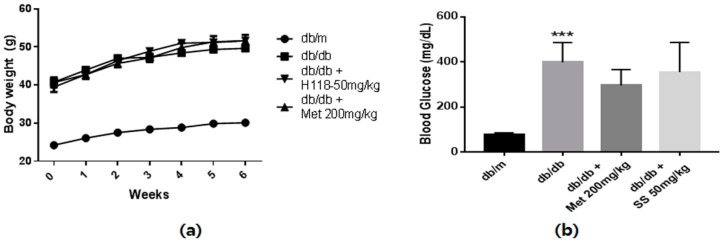

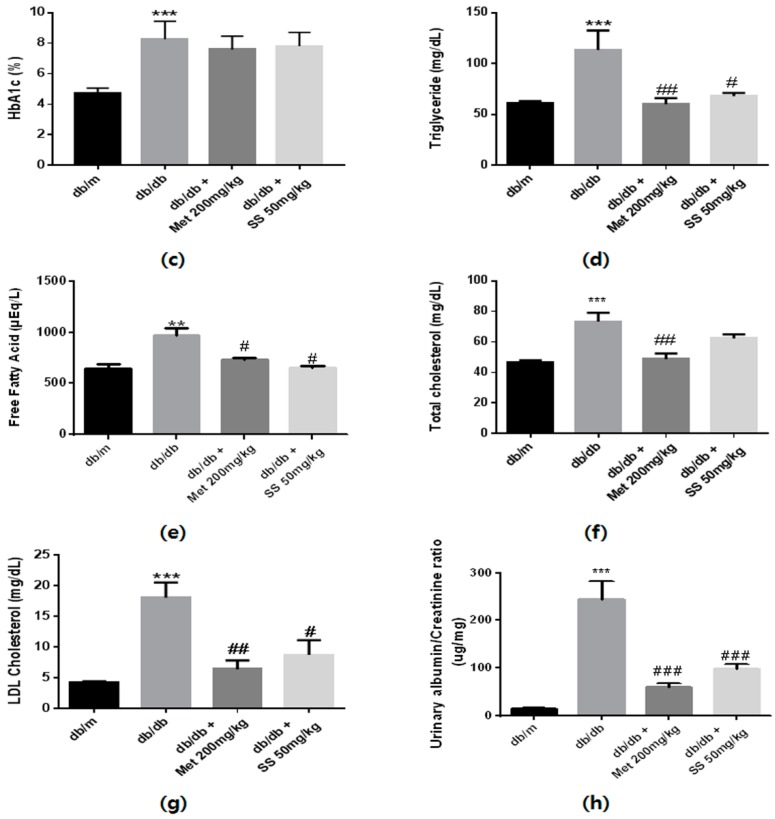
Diabetes parameters and lipid profiles of db/db mice. (**a**) Time course of body weight changes in mice. (**b**) Fasting blood glucose levels of db/m and db/db mice following a 6-week treatment period and after 12 h of fasting. (**c**) Glycated hemoglobin (HbA1c) levels of db/m and db/db mice after a 6-week treatment period. (**d**) Serum triglyceride levels of db/m and db/db mice after a 6-week treatment period and 12 h of fasting. (**e**) Serum-free fatty acid levels of db/m and db/db mice after a 6-week treatment period and 12 h of fasting. (**f**) Serum total cholesterol levels of db/m and db/db mice after a 6-week treatment period and 12 h of fasting. (**g**) Serum low-density lipoprotein (LDL)-cholesterol levels of db/m and db/db mice after a 6-week treatment period and 12 h of fasting. (**h**) Urinary albumin/creatinine ratio of db/m and db/db mice after a 6-week treatment period and 12 h of fasting. Lipid profiles and urine albumin/creatinine ratio were determined using a commercial enzyme-linked immunosorbent assay kit according to the manufacturer’s protocol. Value are means ± standard error of the mean (SEM, ** *p* < 0.01; *** *p* < 0.001 vs. db/m; # *p* < 0.05, ## *p* < 0.01, and ### *p* < 0.001 vs. db/db).

The animals’ body weight was measured every week, and fasting blood glucose and HbA1c levels were determined at day 42 after overnight fasting. As shown in Figure 3a, the body weights of the db/db mouse groups markedly increased compared to that of the normal mice. Moreover, significantly increased fasting blood glucose and HbA1c levels were observed in the db/db mouse groups compared to those in the normal mice (Figure 3b,c). *S*. *suberectus* treatment did not result in significant alterations in body weight, and blood glucose and HbA1c levels. As expected, serum triglyceride, total cholesterol, low-density lipoprotein (LDL)-cholesterol, and free fatty acid (FFA) levels were significantly higher in the db/db control mice than in the normal mice (Figure 3d,e). However, the *S*. *suberectus* extract-treated group showed significantly decreased levels of triglyceride, FFA, and LDL-cholesterol. Moreover, the urinary albumin-to-creatinine ratio (ACR), common clinical indicator to assess the severity of diabetic nephropathy, in the db/db control mice increased compared to that in the normal mice and *S*. *suberectus* extract treatment markedly reversed this change. 

### 2.4. Effects of the S. suberectus Extract on the Expression of CML and RAGE 

**Figure 4:**
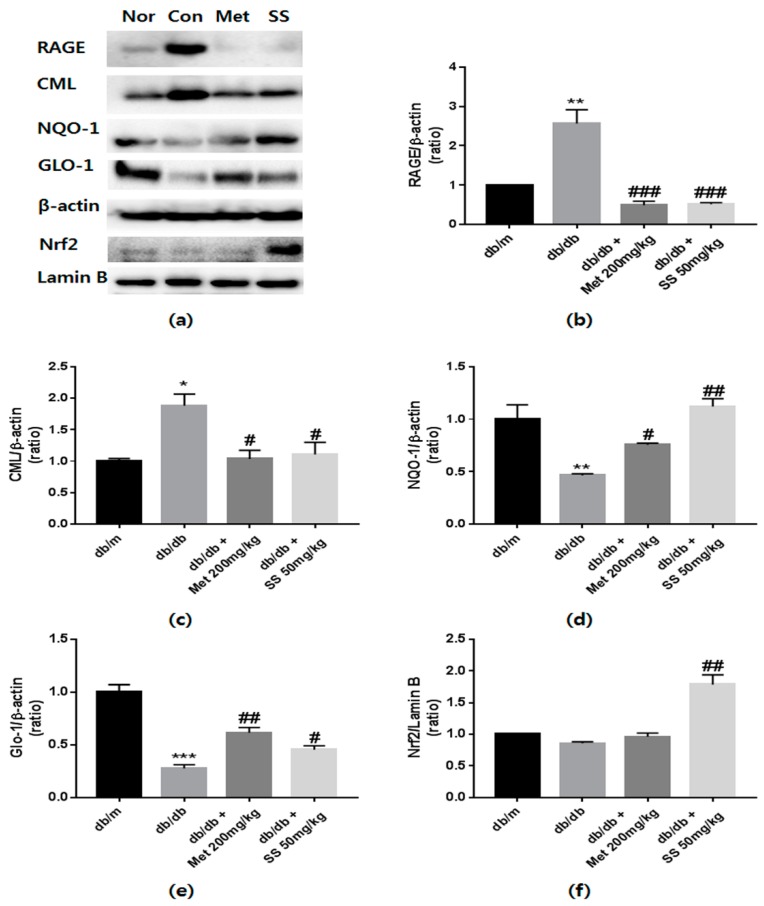
The effects of *Spatholobus suberectus* extract on the protein levels of receptors of advanced glycation end products (RAGE), Nɛ-(carboxymethyl)-lysine (CML), glyoxalase 1 (Glo1), and nuclear factor, erythroid 2 like 2 (Nrf2). (**a**) Representative western blots bands. β-Actin and lamin-B were used as internal controls. Band intensities of (**b**) RAGE, (**c**) CML, (**d**) NQO1, (**e**) Glo1, and (**f**) Nrf2. MET (Metformin) is used for positive control. Tissue samples were pooled and the experiments were repeated three times. Values are shown as means ± standard error of the mean (SEM); (* *p* < 0.05, ** *p* < 0.01 and *** *p* < 0.001 vs. db/m; # *p* < 0.05, ## *p* < 0.01, and ### *p* < 0.001 vs. db/db).

Because increased AGEs levels and AGEs–RAGE interaction induce apoptosis and oxidative stress [21], it was investigated whether treatment with the *S*. *suberectus* extract reduces CML levels and RAGE expression. In diabetic control mice, CML levels and RAGE expression markedly increased compared to those in the normal mice (Figure 4b,c). However, these phenomena were ameliorated by treatment with the *S*. *suberectus* extract. These findings indicated that treatment with the *S*. *suberectus* extract attenuates diabetes-induced kidney damage by regulating RAGE expression and AGEs accumulation.

### 2.5. Effects of the S. suberectus Extract on the Glo1 and Nrf2 Pathway 

Upregulation of Nrf2 and Glo1 expression is a useful approach to protect against AGEs-induced diabetic nephropathy [22]. To examine whether *S*. *suberectus* can regulate the Glo1 and Nrf2 pathway, western blot analysis was performed. As shown in Figure 4d,e, *S*. *suberectus* extract administration resulted in a significant increase in NQO1 and Glo1 expression compared to that in the db/db control mice. Moreover, the *S*. *suberectus* extract-treated group showed significantly increased expression of Nrf2 in nuclei (Figure 4f). Taken together, the findings indicate that the effects of the *S*. *suberectus* extract on the reduction of AGEs accumulation and RAGE expression was attributable to the upregulation of NQO1 and Glo1 proteins through Nrf2 overexpression.

### 2.6. Histology and Immunohistochemistry 

**Figure 5:**
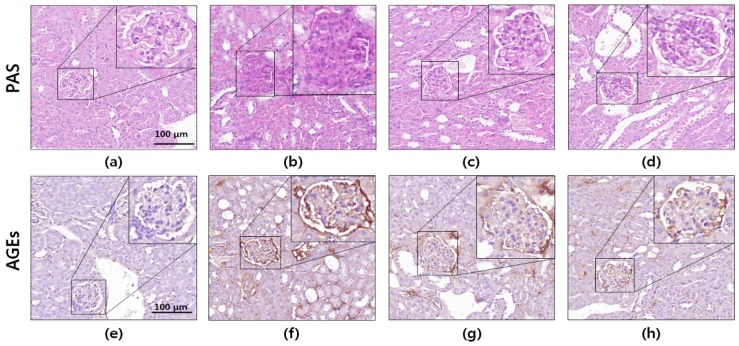
Periodic acid–Schiff (PAS) and immunohistochemical (IHC) staining of db/db mice kidney tissues. Photomicrographs of (**a**–**d**) PAS staining and (**e**–**h**) IHC staining of advanced glycation end products. The images shown are representative of (**a**,**e**) normal mice, (**b**,**f**) db/db control mice, (**c**,**g**) metformin-treated db/db mice, and (**d**,**h**) *Spatholobus suberectus* extract-treated db/db mice. Microscopic images of PAS and IHC staining are presented at 400× magnification.

To evaluate the protective effects of the *S*. *suberectus* extract against renal damage, PAS staining and immunohistochemistry were performed using kidney sections. It is well-known that AGEs cause increased PAS-positive staining [23]. As shown in Figure 5, histochemical staining of the db/db mouse kidney did not show glomerular mesangial expansion. However, significantly elevated PAS-positive staining (glycated protein) was observed in the diabetic mice compared to that in the normal mice. In contrast, oral administration of the *S*. *suberectus* extract attenuated the accumulation of glycogen in the glomerulus.

Immunohistochemical analyses of glomeruli from diabetic control mice exhibited significantly increased AGEs accumulation compared to that in the normal mice. However, the diabetes-induced increase in AGEs accumulation in the glomeruli was markedly reduced by the oral administration of the *S*. *suberectus* extract. These results indicated that the *S*. *suberectus* extract ameliorated hyperglycemia-induced cross-linking of protein and AGEs accumulation.

## 3. Discussion

Diabetes-induced long-term hyperglycemia accelerates AGEs formation and related complications. In the case of the kidney, AGEs accumulation results in thickening of basement membranes, mesangial expansion and hypertrophy, and podocyte loss [4]. Moreover, it is well-known that AGEs-RAGE interaction play an important role in diabetic nephropathy [6]. Thus, preventing the AGEs formation, breaking the AGEs-induced cross-links and downregulation of RAGE represents an effective strategy to prevent the progression of diabetic nephropathy. 

Recent studies have shown that most of phenolic compounds and phenolic compound-rich plant extracts can inhibit AGEs formation and break cross-links [24]. Cheng et al. reported that *S*. *suberectus* contains diverse phenolic compounds [25]. We determined the formononetin amount in *S*. *suberectus* using HPLC and determined the amounts of this components analysis. It was reported that formononetin has an anti-glycation effect [26,27]. For these reasons, we assumed that the *S*. *suberectus* extract might have the ability to inhibit AGEs formation and break down the AGEs-BSA-induced cross-links to collagen. As expected, the *S*. *suberectus* extract showed these anti-glycation effects (Figure 2). These results indicated that the *S*. *suberectus* extract may constitute a promising intervention against diabetic complications. However, further studies are needed to prove the formononetin that mediate the diabetes-induced renal damage of *S*. *suberectus*.

The antidiabetic effect of *S*. *suberectus* in type 1 diabetes is already known [18] but the effect of *S*. *suberectus* on AGEs-induced diabetic nephropathy and the underlying mechanism are yet to be reported. We found that the *S*. *suberectus* extract could attenuate AGEs-induced diabetic nephropathy by using the db/db mouse model. As shown in Figure 3, diabetic control mice showed significantly increased body weight and levels of blood glucose and HbA1c. Treatment with the *S*. *suberectus* extract did not reduce body weight and levels of blood glucose and HbA1c. It is well-known that elevated blood glucose-induced high levels of HbA1c contribute to the onset of diabetic complications. However, the ADDITION study identified a subset of people with high risk for developing T2D that have normal glucose tolerance [28]. Ismail-Beigi et al. also reported that patients with glycemic control with an HbA1c of 7.6% or 6.3% still developed diabetic complications [8]. Moreover, methylglyoxal, a reactive metabolite that is the major precursor in the formation of AGEs, can elevate fatty acid synthase activity [9]. For these reasons, we evaluated the other diabetes-inducing factors such as dyslipidemia, urine ACR, and glycotoxicity in this study. Zhao et al. [18] used 100% ethanol extract of *S*. *suberectus* and C57BL/6j mice, whereas our experiment used 70% ethanol extract and db/db mice. It is well-known that 70% ethanol extraction is good for most of the compounds from polar-nonpolar. However, 100% ethanol extraction is good for most of the polar compounds. *S*. *suberectus* was contained hydrophobic components such as quinones, steroids and procyanidins [15]. These series of components have antiglycation and antidiabetic activity [29,30] and may be more extracted in 100% ethanol extraction. Based on these observations, we cautiously presumed that the enhanced glucose uptake of *S*. *suberectus* in the study by Zhao et al. [18] is attributable to some of the hydrophobic components of the EU extract. However, this discussion may not be conclusive and need to more research.

In this study, treatment with the *S*. *suberectus* extract markedly reduced increased serum triglyceride, FFA, and LDL-cholesterol levels compared to those in the untreated db/db mice (Figure 3). Moreover, it significantly decreased the diabetes-induced increase in ACR. Dyslipidemia plays important roles in the development and progression of diabetic nephropathy [31]. Significantly increased level of triglycerides and total cholesterol were observed in patients with diabetic nephropathy [32]. Moreover, high levels of triglycerides, total cholesterol, and LDL-cholesterol contribute to albuminuria [33]. Therefore, reducing the serum levels of FFAs, triglycerides, and LDL-cholesterol could be a useful therapeutic approach for treating diabetes nephropathy. Thus, on the basis of our results, we proposed that *S*. *suberectus* may prevent progression of diabetic nephropathy by reducing dyslipidemia.

Many previous studies have reported that increased levels of AGEs are a potent inducer of mitochondrial ROS production [34]. ROS signaling is one of the downstream signaling of AGEs-RAGE interaction and it is known to lead to renal cell death [35]. Glomerular sclerosis and reduced nitric oxide production were induced by AGEs-RAGE interaction, thereby leading to tubulointerstitial fibrosis [36]. For this reason, reducing AGEs accumulation and RAGE expression is an effective strategy to treat the diabetic complications. Nrf2 is well-known as transcription factor for antioxidant enzyme and protect against renal damage [37]. Furthermore, it is a redox regulator that integrates with antioxidant response elements (AREs) in promoter regions of antioxidant genes such as HO-1 and NQO1 [38]. Moreover, Nrf2 has been reported to increase Glo1-ARE transcriptional levels [39]. In this study, we found increased CML levels and RAGE expression and simultaneously reduced Nrf2 expression in db/db mouse kidney (Figure 4). However, *S*. *suberectus* extract treatment upregulated Nrf2 expression and reduced CML levels and RAGE expression. Moreover, *S*. *suberectus* extract treatment resulted in an increased expression of NQO1, a multifunctional antioxidant enzyme, and Glo1. These results suggested that *S*. *suberectus* extract treatment reduced AGEs accumulation and AGEs-RAGE interaction by upregulating Glo1 and NQO1 expression via the Nrf2 pathway in the kidney, thereby ameliorating diabetic nephropathy.

Previously studies have reported the antidiabetic effect of *S*. *suberectus* [18]; however, the protective effects of *S*. *suberectus* against glucotoxicity and diabetes-induced renal damage have not yet been reported. In this study, it was found that *S*. *suberectus* extract treatment significantly inhibited AGEs formation and AGEs cross-links. Moreover, it attenuated dyslipidemia, glucotoxicity, and histological changes through the Glo1 and Nrf2 pathway. Taken together, the findings indicate that the *S*. *suberectus* extract might protect the kidney against diabetes-induced renal damage. Further studies are needed to identify the main active compounds in *S*. *suberectus* that mediate diabetic nephropathy.

## 4. Materials and Methods 

### 4.1. Materials

Glucose, fructose, BSA, sodium azide, metformin, and a phosphatase inhibitor cocktail were purchased from Sigma (St. Louis, MO, USA). Antibodies against RAGE, Nrf2, lamin-B, β-actin, Glo1, and NQO1 were purchased from Cell Signaling Technology (Danvers, MA, USA). The anti-AGEs antibody was obtained from Abcam (Cambridge, MA, USA). The anti-Nɛ-(carboxymethyl)-lysine (anti-CML) antibody was obtained from R&D Systems (Minneapolis, MN, USA) and secondary antibodies were purchased from Thermo Fisher Scientific (Waltham, MA, USA). Nuclear/Cytosol Fraction Kit was obtained from Biovision (Milpitas, CA, USA).

### 4.2. Sample Preparation

*S*. *suberectus* stem (voucher specimen No. H-434) was purchased from Gyeongdong Market (Seoul, Korea) and deposited at the Korea Food Research Institute. The stem was extracted with 70% ethanol by sonication for 1 h. The extract was filtered, evaporated, and then lyophilized. The final extract was stored at −20 °C until used and the yield was 6.5%. 

### 4.3. HPLC analysis

HPLC was performed on a Waters system (Waters Corp., Milford, MA, USA) consisting of a separation module (e2545) with an autosampler (2707) and a photodiode array detector (2998). A Zorbax Eclipse XDB-C18 column (250 × 4.6 mm; particle size, 5 µm; Agilent Technologies, Santa Clara, CA, USA) was used for the chromatographic separation, and the mobile phase was composed of distilled water (solvent A) and acetonitrile (solvent B). The mobile phase flow rate was 1 mL/min and the gradient was as follows: 0 min, 85% A; 40 min, 35% A. UV absorbance was monitored from 190 to 400 nm, and a qualitative analysis was conducted at 254 nm.

### 4.4. Inhibitory Effects of the S. suberectus Extract on AGEs Formation 

BSA (10 mg/mL) was incubated with glucose and fructose (25 mM) in phosphate-buffered saline, pH 7.4, in the presence or absence of *S*. *suberectus* extract (10, 50 and 100 μg/mL) with 0.02% sodium azide. Reaction mixtures were incubated at 37 °C for 14 days. Stock solution of *S*. *suberectus* was dissolved in DMSO at 100 mg/mL. Inhibitory effects of *S*. *suberectus* on AGEs formation were detected using fluorescence at an excitation/emission wavelength of 350/450 nm with a microplate reader (Molecular Devices, Sunnyvale, CA, USA). 

### 4.5. AGEs Cross-Link-Breaking Ability of the S. suberectus Extract 

The AGEs cross-link–breaking assay was performed according to the method described by Lee et al. [40] with slight modifications. For the assay, 1 mg/mL peroxidase-labeled AGEs-BSA was pre-incubated on collagen-coated 96-well plates at 37 °C for 4 h. After 1 mM AG or *S*. *suberectus* (10, 50 and 100 μg/mL) was added and incubation was performed for 24 h. The cross-link–breaking ability of the extract was detected using tetramethylbenzidine substrate and expressed as the percentage decrease in optical density. 

### 4.6. Animals

Six-week-old male C57BL/KsJ db/db mice and db/m mice were purchased from Joongang Laboratory Animal Inc., (Seoul, Korea). The animals were kept in a thermoregulated and humidity-controlled animal facility on a 12-h/12-h light/dark cycles and given food and water ad libitum. At 7 weeks of age, the mice were divided at random into four treatment groups (*n* = 8): (1) normal mice (normal group; C57BL/KsJ-db/m mice); (2) diabetes control mice (control group; db/db mice); (3) metformin-treated mice (MET group; db/db mice orally administered with 200 mg/kg metformin); and (4) *S*. *suberectus* extract-treated mice (db/db mice orally administered with 50 mg/kg *S*. *suberectus* extract). The *S*. *suberectus* extract was dissolved in distilled water. We orally administered the extract or metformin, and the other group was given the same amount of vehicle for 6 weeks. All animal experiments conformed to the guidelines for Animal Care and Use Committee of the Korea Food Research Institute (approval number KFRI-M-17026).

### 4.7. Blood and Urine Analysis 

Prior to sacrificing the animals, fasting blood glucose and HbA1c levels were determined by using a glucometer (Accu-chek, Roche Diagnostics, Basel, Switzerland) and an HbA1c analyzer (Infopia Inc., Anyang, Korea) as described in the manufacturer’s instructions. To quantify diabetes-induced dyslipidemia, blood samples were collected and serums samples obtained were frozen at −80 °C until biochemical analysis. The serum levels of triglyceride, FFA, total cholesterol, and LDL cholesterol were determined using a commercial enzyme-linked immunosorbent assay kit (Cell Biolabs, Inc., Beverly, MA, USA) according to the manufacturer’s protocol. Urine samples were obtained to determine the ACR using a metabolic cage for 24 h and stored at −20 °C prior to sacrificing the animals. The urinary ratio of albumin/creatinine was measured using an ACR assay kit (Biovision, Milpitas, CA, USA) according to the manufacturer’s instructions.

### 4.8. Western Blotting Analysis

Total proteins from the kidney were extracted with PRO-PREPTM (iNtRON Biotechnology, Seongnam, Korea) containing phosphatase inhibitor and nuclear and cytosol lysates were isolated using a Nuclear/Cytosol Fractionation Kit according to the manufacturer’s instructions. Protein samples were loaded on 10% sodium dodecyl sulfate polyacrylamide gel electrophoresis (SDS-PAGE) gels and then transferred onto polyvinylidene fluoride (PVDF) membranes. The membranes were blocked in 5% skim milk. After blocking, the membranes were incubated with the primary antibodies overnight at 4 °C and then the secondary antibodies were incubated for 1 h. Western blot bands were observed using an enhanced chemiluminescence solution with a ChemiDoc XRS+ imaging system (Bio-Rad, Hercules, CA, USA).

### 4.9. Histology and Immunohistochemistry 

Kidney tissues fixed in 4% formaldehyde were embedded in paraffin. Sections (thickness, 4 μm) were stained with periodic acid–Schiff (PAS) stain for histological analysis. To evaluate AGEs accumulation in kidney glomeruli, 3-μm-thick sections were used. Antigen retrieval was performed using 20 μg/mL proteinase in phosphate buffered saline (PBS) for 20 min at 37 °C. To quench the endogenous peroxidase activity, tissue sections were incubated with 3% H_2_O_2_ in PBS for 15 min. The sections were blocked using 1% horse serum in PBS and then incubated overnight at 4 °C with primary antibodies against AGEs (1:500). Thereafter, the sections were incubated with the secondary antibodies (1:200) for 20 min at room temperature and then incubated with Vectastain ABC reagent (Vector Laboratory, Piscataway, NJ, USA) for 30 min. 3,3′-Diaminobenzidine (Vector Laboratory, Piscataway, NJ, USA) was used to determine the immunohistochemical development, and the sections were counterstained with hematoxylin. Pannoramic 250 Flash III slide scanner (3DHistech, Ltd., Budapest, Hungary) and CaseViewer software (3DHistech, Ltd.) were used for scanning and analyzing sections. We randomly selected ten glomeruli per mouse for identification of renal morphometric changes. 

### 4.10. Statistical Analysis

The results of statistical analyses are presented as mean ± SEM values and comparisons between groups were performed using one-way ANOVA followed by Bonferroni’s test. A *p* value < 0.05 was considered statistically significant. All data were analyzed using GraphPad Prism 5 (GraphPad Software, Inc., San Diego, CA, USA). 

## 5. Conclusions

Upregulation of NQO1 and Glo1 signaling improves AGEs-induced glucotoxicity and reduces RAGE expression. Moreover, dyslipidemia, oxidative stress and albumin/creatinine ratio were improved by *S*. *suberectus* extract treatment. This is predominantly due to increased Nrf2 activation; thus, *S*. *suberectus* extract treatment ameliorates diabetes-induced renal damage. These findings suggest that the *S*. *suberectus* extract could be a potential supplement for the prevention or treatment of diabetic nephropathy. 

## Abbreviations

AGEs	advanced glycation end products
ARE	antioxidant responsive element
AG	aminoguanidine
ALT-711	alagebrium
CML	Nɛ-(carboxymethyl)-lysine
BSA	bovine serum albumin
Glo1	glyoxalase 1
MET	metformin
Nrf2	nuclear factor erythroid 2-related factor 2
NQO1	NADPH quinine oxidoreductase 1
RAGE	receptor for AGEs
ROS	reactive oxygen species
PAS	periodic acid–Schiff
IHC	immunohistochemistry
